# Periodontitis as a risk factor for organic erectile dysfunction: A case-control study in a sub-Saharan population

**DOI:** 10.34172/japid.2023.021

**Published:** 2023-11-08

**Authors:** Abdoulaye Diouf, William Ndjidda Bakari, Marie Hélène Sounlin, Ahmad Moustapha Diallo, Daibel Thiam, Mouhamadou Lamine Guirassy, Adam Seck Diallo, Henri Michel Benoist

**Affiliations:** Periodontology Department, Faculty of Medicine, Pharmacy and Odontology, University Cheikh Anta Diop of Dakar, Senegal

**Keywords:** Endothelial dysfunction, Erectile dysfunction, Periodontitis, Sexual dysfunction

## Abstract

**Background.:**

This study investigated the association between periodontitis and organic erectile dysfunction (ED) in a sub-Saharan population.

**Methods.:**

This multicenter analytical study lasted from April to September 2021. A total of 114 patients (38 cases and 76 controls) were recruited and matched on age, diabetes, and smoking status. Medical history and ED were recorded, as well as the plaque index, bleeding index, maximum interdental clinical attachment loss (CALmax), maximum probing depth, clinically detectable furcation involv ement, number of teeth in the mouth, number of teeth lost for periodontal reasons, and tooth mobility. The analysis was performed with SPSS 20.0 with a significance threshold set at 5%.

**Results.:**

The two study groups were comparable regarding sociodemographic characteristics. Periodontitis was present in 76.31% of cases and 75% of controls without a significant difference (*P*=0.878). Logistic regression showed a significant association between high blood pressure and ED with an OR=4.78 (95% CI: 1.80‒12.70). Periodontitis was not associated with ED (OR=1.52, 95% CI: 0.55‒4.16); however, severe periodontitis was significantly associated with severe ED (OR=1.44, 95% CI: 1.11‒1.85, and OR=1.68, 95% CI: 1.15‒2.44, respectively for CALmax and tooth loss).

**Conclusion.:**

Within the limits of this study, periodontitis was not associated with organic ED. However, the severity of periodontal disease significantly increased in patients with organic ED.

## Introduction

 Periodontitis is a chronic polymicrobial and inflammatory infection initiated by an accumulation of dysbiotic plaque known as biofilm above and below the marginal gingiva, within which bacterial products cause a chronic, ongoing inflammatory response.^1^ Periodontitis affects tooth-supporting tissues, extends beyond the oral sphere, and is linked to several systemic diseases.^2^

 According to the National Institutes of Health, erectile dysfunction (ED) is the consistent or recurrent inability to achieve and/or maintain a penile erection sufficient for satisfactory sexual performance. It is mainly due to organic dysfunction, psychological factors, or both, and its prevalence increases with age.^3-5^ The classification of organic ED depends on the underlying pathophysiological mechanism(s), including vascular, neurogenic, structural, and hormonal causes.^6^ It is a significant public health problem, responsible for increased stress and altered self-confidence, affecting patients’ quality of life and relationships with sexual partners.^5^ A growing body of evidence points to a possible association between periodontitis and ED, although controversy persists.^7^ Indeed, Uppal et al^8^ found an association between the presence of periodontitis and ED, and Oğuz et al^9^ suggested chronic periodontitis as an additional risk factor for ED.In contrast, Sharma et al.^10^ did not find a significant association between ED and the presence of periodontitis. Furthermore, recent systematic reviews by Farook et al^11^ and Lecaplain et al^12^ showed an association between periodontitis and ED. However, the effect of confounding factors and recall bias remain major limitations to the studies considered in these reviews. Moreover, the biological mechanisms linking these two conditions remain poorly understood. Bacteriological and inflammatory theories have suggested that after entering the bloodstream, the bacterial periodontopathogens involved in the association between these two diseases cause bacteremia and inflammation at a distance from the oral cavity by bacterial translocation.^5^ These bacteria can invade, penetrate, and persist in vascular endothelial cells.^13-15^ Bacteremia causes an inflammatory syndrome resulting in an increase in pro-inflammatory mediators, including interleukins (IL-1 and IL-6) and tumor necrosis factor-α (TNF-α). Stimulation of endothelial cells by these pro-inflammatory mediators leads to endothelial dysfunction (EDys).^14^ ED appears to be the emerging part of a larger EDys affecting all vascular territories to varying degrees and falsely a locoregional vascular disease limited to the corpora cavernosa alone.^16^ A role for oxidative stress has also emerged. In the presence of this stress, body cells are altered, and their functions are modified.^17^ Periodontitis causes an attenuation of the total antioxidant capacity of plasma, resulting in low-grade systemic inflammation.^14^ It has also been proposed that periodontitis contributes to the etiology of ED by increasing the production of reactive oxygen species (ROS) in tissues. However, nitric oxide (NO), the main neuromodulator responsible for intrapenile smooth muscle relaxation, can combine with various ROS, which reduces its bioavailability, thus enhancing EDys and altering mechanisms associated with muscle contraction.^14,18^

 The understanding of a possible interaction between periodontitis and ED opens up new horizons in treating this disease. Even though epidemiological studies have been conducted, they have limitations that often hinder interpreting their results (failure to consider confounding factors, small sample size, and lack of group heterogeneity). ED is a public health issue, both in terms of its increasing prevalence and the psychological and social consequences for affected patients.^19^ To our knowledge, no clinical studies have been published investigating the relationship between periodontitis and ED in sub-Saharan Africa. Therefore, we aimed to investigate the association between periodontitis and organic ED in a sub-Saharan Senegalese population.

## Methods

###  Study population

 This multicenter case‒control study was conducted in the Urology/Andrology Departments of the Aristide le Dantec Hospital (HALD), the Military Hospital of Ouakam (HMO), and the Abass Ndao Hospital Center (CHAN) for both cases and controls. This study lasted from April 09 to September 15, 2021 (5 months). The clinical event was organic ED. Controls were free of ED and had not been previously diagnosed with ED.

 Sampling was consecutive. The cases and controls were recruited according to patient record files of newly diagnosed patients. The sample size was determined for a two-tailed t-test by G*Power 3.5.1 software, with a size effect of 3, an alpha risk of 0.05, 1-Beta equal to 0.8, and a ratio of 2 controls for 1 case. Then, the sample consisted of 38 patients with ED and 76 controls matched on age, diabetes, and smoking status, totaling 114 patients.

###  Inclusion criteria and case definition

 Any patient with a clinical diagnosis of ED aged 18‒80, consulting in the urology-andrology services, having engaged in sexual activity in the six months before the survey, and consenting to participate in the study was included. Patients who chose to leave the study for various reasons were excluded.

 ED diagnosis was established using the Simplified International Index of Erectile Function (IIEF-5) self-assessment questionnaire, which assesses sexual function over the past six months. ED was considered severe when the score range was 5‒10, moderate between 11 and 15, and mild between 16 and 20. Between 21 and 25, erectile function was normal, and between 1 and 4, the score was considered uninterpretable. However, illiterate patients who could not self-administer the questionnaire were classified as mild ED if there was a rigidity defect with the possibility of penetration, moderate ED in those with a rigidity defect and inability to penetrate the partner, and severe ED if no erection was present. A dental clinical examination was performed on all the patients.

 Patients with at least 15 natural permanent teeth in the mouth (excluding wisdom teeth and teeth requiring extraction due to dental caries), with detectable interdental clinical attachment loss (CAL) on at least two non-adjacent teeth or buccal or oral CAL ≥ 3 mm with pocketing > 3 mm detectable at ≥ 2 teeth which could not be ascribed to non-periodontal causes (endo-periodontal lesions, vertical fractures, impacted wisdom teeth, cavities, or restorations) were considered periodontitis patients.^20^ Periodontitis was defined as stage 1 or 2 when CAL < 5 mm and a maximum probing depth (PDmax) of < 6 mm. It was considered stage 3 or 4 in cases of CAL > 5 mm and PDmax ≥ 6 mm. For stage 3 or 4, the number of missing teeth for periodontal reasons, furcation involvement, and tooth mobility were recorded.

###  Data collection

 Patients fulfilling inclusion criteria had their sociodemographic data, comorbidities, history of ED, and oral hygiene habits collected. They subsequently underwent a periodontal examination grouping assessment of plaque index, bleeding on probing index (BOP), maximum interdental clinical attachment loss (CALmax), maximum probing depth (PDmax), clinically detectable furcation damage, number of teeth in the mouth, number of teeth lost for periodontal reasons and the number of teeth with Mühlemann (1954) mobility ≥ 2. Periodontal probing was carried out using a Williams periodontal probe. Pocket depth represents the height between the top of the gingival margin and the bottom of the pocket. Interdental attachment loss is the distance from the cementoenamel junction to the bottom of the periodontal pocket, measured at the proximal (mesial and distal) surfaces of the teeth. All the remaining teeth were considered except wisdom teeth and teeth with total coronal decay indicating extraction.

 A clinical examination form had been pre-established, and patients were recruited during urological consultations. Four calibrated, masked examiners performed periodontal examinations.

###  Statistical analyses

 Data were analyzed using Statistical Package for Social Sciences (SPSS) version 20.0. Categorical variables were expressed as a percentage and frequency. The mean and standard deviation of the mean were calculated, as well as the minimum and maximum for quantitative variables. Chi-square or Fisher’s exact tests was used to investigate the association between categorical variables, while Student’s or Wilcoxon’s test was used for quantitative variables. Binary logistic regression was performed to investigate the relationship between organic erectile and some potential associated factors. Matching and multivariate analysis were considered to manage confounding factors. The significance level was set at 5%.

## Results

**Figure 1:**
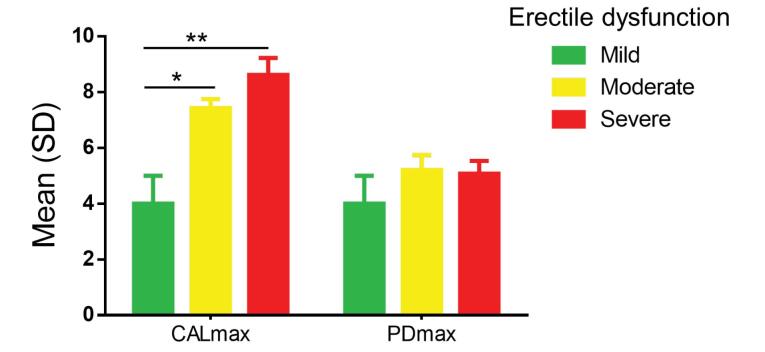
Distribution of CALmax and PDmax according to erectile dysfunction severity. Analysis of variances of CALmax and PDmax with erectile dysfunction’s severity degree. ANOVA intergroup for CALmax: F = 2.64, *P* = 0.03*. ANOVA intergroup for PDmax: F = 0.30, *P* = 0.94. SD, standard deviation; CALmax, maximum interdental clinical attachment loss; PDmax, maximum probing depth. Mann-Whitney test. * P < 0.05; **P < 0.01

 A total number of 114 participants were recruited (38 cases and 76 controls). Depending on whether recruitment was done at the HALD, the HMO, or the CHAN, the cases were 8 (21.05%), 8 (21.05%), and 22 (57.89%), respectively. The controls were 28 (36.84%) at HALD, 17 (21.79%) at HMO, and 31 (40.78%) at CHAN. The two groups were similar regarding sociodemographic characteristics and clinical history (Table 1). However, there were more cases with a history of hypertension (*P* < 0.01). The moderate form of ED comprised 50% of the cases. Concerning periodontal-related parameters (Table 2), the plaque index (85.41 ± 25.74% and 85.17 ± 25.21%; *P* = 0.54) and BOP (62.24 ± 9.89 and 56.18 ± 35.88; *P* = 0.38) were higher than 50% in both cases and controls without a statistically significant difference between the two groups, while PDmax was > 5 mm in both groups without significant difference. CALmax was greater in the cases than in the controls (7.72 ± 2.05 mm and 6.86 ± 1.9 mm, respectively; *P* = 0.04). Similarly, the severity of periodontal damage was greater according to the severity of ED (ANOVA for CALmax according to ED severity, *P* = 0.03; Figure 1). Moreover, CALmax was associated with severe ED:OR = 1.308 with a 95% confidence interval (CI) of 1.00‒1.70 (Table 3). The number of teeth lost due to periodontal disease was higher (2.11 ± 3.15 and 0.76 ± 2.04; *P* = 0.01) in cases than in controls. Periodontitis was found in a comparable proportion of cases and controls [29 (76.31%) and 57 (75%); *P* = 0.87]. According to logistic regression (Table 4), hypertension was statistically associated with ED (OR = 4.78 and CI: 1.80‒12.70; *P* = 0.002). Periodontitis resulted in a non-significant increase in the odds ratio for ED of 1.524 (95% CI: 0.55‒4.16; *P* = 0.411) despite the association between severe CAL and severe ED (Table 3).

**Table 1:** Sociodemographic and clinical characteristics of the study population

**Variables**	**Cases (n=38)**	**Controls (n=76)**	***P***** value**
Mean age ± SD	60.45 ± 10.04	59.04 ± 10.14	0.48
Marital status			
Single	0 (00)	6 (7.89)	0.24
Divorce	2 (5.26)	2 (2.63)	
Married	36 (94.73)	67 (88.15)	
Widow	0 (00)	1 (1.31)	
Occupation			0.34
Student	0 (00)	1 (1.31)	
Jobless	15 (39.47)	21 (27.53)	
Private/public	14 (36.84)	40 (52.63)	
Daily worker	9 (23.68)	14 (18.42)	
Diabetes mellitus			1.00
Yes	11 (28.9)	22 (28.9)	
No	27 (71.1)	54 (71.1)	
Tobacco			1.00
Yes	3 (7.9)	6 (7.9)	
No	35 (92.1)	70 (92.1)	
Alcohol			**-**
Yes	0 (0)	9 (11.8)	
No	38 (100)	67 (88.2)	
Hypertension			0.00*
Yes	19 (50)	16 (21.1)	
No	19 (50)	60 (78.9)	
Body mass index (kg/m^2^)	24.98 ± 2.44	25.02 ± 1.89	0.71
Erectile dysfunction			-
Mild	5 (13.2)	-	
Moderate	19 (50)	-	
Severe	14 (36.8)	-	
No	-	76 (100)	
Site of recruitment			0.16
HALD	8 (21.05)	28 (36.84)	
HMO	8 (21.05)	17 (21.79)	
CHAN	22 (57.89)	31 (40.78)	

SD, standard deviation; HALD, Aristide Le Dantec Hospital; HMO, Ouakam Military Hospital; CHAN, Abass Ndao Hospital Center. * *P* < 0.05.

**Table 2:** Periodontitis-related characteristics

**Variables**	**Case (n=38)**	**Control (n=76)**	***P***** value**
Plaque index (mean ± SD)	85.41 ± 25.74	85.17 ± 25.21	0.54
BOP (mean ± SD)	62.24 ± 39.89	56.18 ± 35.88	0.38
CALmax (mean ± SD)	7.72 ± 2.05	6.86 ± 1.9	0.04*
PDmax (mean ± SD)	5.07 ± 1.79	5.47 ± 1.65	0.27
Furcation involvement			
Yes	4 (10.52)	5 (6.57)	0.46
No	34 (89.47)	71 (93.42)	
Number of teeth (mean ± SD)	25.58 ± 5.19	27.51 ± 4.06	0.03*
Tooth loss (mean ± SD)	2.11 ± 3.15	0.76 ± 2.04	0.01*
Tooth mobility (mean ± SD)	0.50 ± 0.95	0.62 ± 2.15	0.74
Periodontitis			
Yes	29 (76.31%)	57 (75%)	0.87
No	9 (23.68%)	19 (25%)	
Stage 1 or 2			
Yes	2 (5.26)	7 (9.21)	0.44
No	27 (75.05)	50 (65.78)	
Stage 3 or 4			
Yes	27 (75.05)	50 (65.78)	0.71
No	2 (5.26)	7 (9.21)	

SD: standard deviation; BOP, bleeding on probing; CALmax, maximum interdental clinical attachment loss; PDmax, maximum probing depth. * *P* < 0.05.

**Table 3:** Multivariate analysis with binary logistic regression: severe erectile dysfunction and associated factors

**Variables**	**B**	**SE**	**Wald**	***P***** value**	**Exp(B)**	**95% CI**
Age	-0.046	0.048	0.887	0.346	0.955	0.87‒1.05
High blood pressure	1.892	0.836	5.117	0.024*	6.632	1.28‒34.16
Body mass index	-0.208	0.186	1.253	0.263	0.812	0.56‒1.17
Number of teeth	0.143	0.131	1.183	0.277	1.153	0.89‒1.49
CALmax	0.268	0.134	4.016	0.045*	1.308	1.00‒1.70
Tooth loss	0.518	0.192	7.252	0.007**	1.68	1.15‒2.44
Constant	-1.564	6.226	0.063	0.802	0.209	

B, coefficient for the constant; SE, standard error around B coefficient; Wald, Wald chi-square test; Exp(B), exponentiation of the B coefficient; CI, confidence interval; CALmax, maximum interdental clinical attachment loss. Degree of freedom = 1; * *P* < 0.05, ** *P* < 0.01.

**Table 4:** Multivariate analysis with a binary logistic regression: erectile dysfunction and associated factors

**Variables**	**B **	**SE**	**Wald**	***P***** value**	**Exp(B)**	**95% CI**
Age	-0.012	0.023	0.282	0.595	0.988	0.94‒1.03
Tobacco^a^	0.387	0.796	0.236	0.627	1.472	0.30‒7.00
Diabetes^a^	-0.073	0.479	0.023	0.879	0.929	0.36‒2.37
High blood pressure	1.565	0.498	9.873	0.002**	4.784	1.80‒12.70
Periodontitis^a^	0.421	0.513	0.675	0.411	1.524	0.55‒4.16
Constant	-0.830	1.400	0.351	0.553	0.436	

B, coefficient for the constant; SE, standard error around B coefficient; Wald, Wald chi-square test; Exp(B), exponentiation of the B coefficient; CI, confidence interval. Degree of freedom = 1; ** *P* < 0.01; ^a^ Categorial variable ‘yes’.

## Discussion

 This study investigated the association between periodontitis and organic ED in a case‒control study. Although the severity of periodontal disease (CALmax and the number of teeth missing due to periodontal disease) was associated with ED, there was not a statistically significant association between periodontitis and organic ED (OR = 1.5, 95% CI: 0.4‒4.1). Numerous preclinical studies have indicated that ED is a common medical condition in males aged ≥ 50, which may increase the likelihood of periodontitis in these individuals.^7^ Matsumoto et al^21^ reported an average age of 50.9 ± 16.6 years for ED cases in a Japanese population, while Martín-Amat et al^22^ reported an average age of 53 ± 9 years for cases and 53 ± 8 years for controls in their study of a Spanish population. The average age of the study population was country-specific, according to Diallo et al,^23^ who found an average age of 45 ± 5 years of patients with ED in Senegal.

 The association between periodontitis and ED remains controversial in the literature. In this study, the prevalence of periodontitis in patients with ED was the highest in those with moderate ED (48%). The prevalence of chronic periodontitis was the highest in patients with severe ED (81.8%) in a study by Sharma et al.^10^ Similarly, Zadik et al^24^ reported that chronic periodontitis was significantly more prevalent in men with mild and moderate-to-severe ED than in men without ED.Not all participants with ED had periodontitis, and the diagnostic methods varied from study to study. Periodontitis was considered an adjustment variable by Martín et al^25^ or a confounding variable for Uppal et al.^8^ Interdental CAL is the main criterion for assessing the severity of periodontal diseases. In the present study, a patient was considered to have periodontitis according to criteria from the new classification of periodontal diseases.^20^

 A significant association between CALmax and ED (*P* = 0.045) was observed; the severity of CALmax impairment was statistically associated with the severity of ED (*P* = 0.037). Sharma et al^10^ also found a continuous increase in mean CAL with the severity of vasculogenic ED. In contrast, Uppal et al^8^ found a positive correlation between ED severity, blood pressure, and alveolar bone loss. Similarly, we found a significant association between ED and the number of teeth lost for periodontal reasons (*P* = 0.016). Periodontal tooth loss is a sign of anterior pathological mobility, which is one of the main symptoms of periodontitis. Fujitani et al^26^ found a positive relationship between tooth mobility and EDys, with the latter recognized as one of the underlying pathophysiological features of ED. We may, like Oğuz et al^9^ say there is a link between some clinical periodontal parameters and ED. Twenty-nine patients (76.31%) with organic ED versus 57 (75%) without organic ED had periodontitis. According to multivariate analysis, periodontitis resulted in a 1.5-fold increase in the odds ratio of having ED, i.e., 50% more likely than those without periodontitis, despite that no statistically significant association was found here between periodontitis and organic ED (OR = 1.5; 95% CI: 0.4-4.1). However, we noted that periodontitis was milder in controls than in cases. Conversely, it was more severe in cases than in controls. According to Keller et al^27^ and Garcia,^28^ there is a significant association between ED and being diagnosed with chronic periodontitis. Eltas et al,^29^ when assessing the relationship between ED severity and periodontal treatment, noted that ED severity improved after periodontal treatment. Keller et al,^27^ through their study of the Taiwanese population, showed evidence of an association between gingivectomy or periodontal flap surgery and ED. This finding is evidence that the association between periodontitis and ED is mediated by inflammation associated with periodontitis.

 As strengths, this multicenter study is, to our knowledge, one of the first in sub-Saharan Africa in general. The cases were incidental, and the controls were selected in the same department to minimize selection bias. A restriction was made on common confounding factors (age, diabetes, and smoking). Examiners were masked, and an appropriate statistical method was used to control for confounding factors. However, some flaws must be mentioned. To determine the severity of ED, the IIEF-5 questionnaire was used, which has limitations both in its application and in the diagnosis of organic ED, where Doppler ultrasound is more appropriate. Moreover, this questionnaire was not used in all the patients because some participants were illiterate, and some had difficulties understanding and completing it. Thus, the failure to carry out radiographic, biological, and endothelial function examinations was a limitation in the performance of this study to ascertain diagnoses.

 While hoping that further studies will be conducted on this subject, given periodontitis and organic ED are both major public health problems, it would be wise for urologists to inform patients with organic ED of the possibility of an association between the two conditions and to urge them to consult a dentist for oral health management. Dentists should also follow suit by recommending patients > 40 to be screened for organic ED by a urologist.

## Conclusion

 Within the limitations of this study, periodontitis was not associated with an increase in the odds ratio of ED. However, the severity of periodontal disease was associated with severe forms of ED.

## Acknowledgments

 We express our gratitude to Mrs. Kodia Debora for her valuable contribution in enhancing the clarity of the document.

## Competing Interests

 The authors declare that they have no competing interests.

## Data Availability Statement

 The datasets used and analyzed during the current study are available from the corresponding author upon reasonable request.

## Ethical Approval

 This study was designed and reported according to the STROBE statement and approved by authorization (No. 239/05/2022) of the Ethics Committee of the Faculty of Medicine, Pharmacy and Odontology-Stomatology of the University Cheikh Anta Diop of Dakar. The Helsinki Declaration for Health Research was respected, and informed consent was signed by each patient before inclusion in the study, as well as the guarantee and respect of anonymity and confidentiality of collected data.

## Funding

 The authors declared no specific grant for this research from any funding agency in the public, commercial, or not-for-profit sectors.
